# Effects of Mixing Combination on Soil Physicochemical Property and Microbial Community in Alfalfa–Grass Mixtures After Seven Years of Establishment

**DOI:** 10.3390/microorganisms14040737

**Published:** 2026-03-26

**Authors:** Jiaojiao Zhang, Xiaojuan Wu, Junyu Zhang, Huimin Yang

**Affiliations:** 1 College of Pastoral Agriculture Science and Technology, Lanzhou University, Lanzhou 730020, China; zhangjj2023@lzu.edu.cn (J.Z.); wuxj20@lzu.edu.cn (X.W.); zhjunyu2024@lzu.edu.cn (J.Z.); 2 State Key Laboratory of Herbage Improvement and Grassland Agro-Ecosystems, Lanzhou University, Lanzhou 730020, China; 3 Qingyang National Field Scientific Observation and Research Station of Grassland Agro-Ecosystems, Lanzhou University, Qingyang 745000, China

**Keywords:** alfalfa–grass mixture, forage yield, soil physicochemical property, soil microbial community structure, soil health

## Abstract

Cultivation of perennial mixtures has emerged as an efficient way to produce a large amount of forage, supporting a sustainable livestock industry. The stability and sustainability of forage production is largely controlled by soil health. However, variation in soil health in perennial mixtures still needs further investigation under diverse conditions. Clarifying the relationships between soil physicochemical properties and microbial community is of great importance in better understanding soil health in perennial cultivated grasslands. The effects of mixing combination of alfalfa with timothy or smooth bromegrass on soil health were evaluated through comparing soil nutrients, enzyme activities, microbial community, and forage yield in alfalfa–grass mixtures and corresponding monocultures after seven years of establishment. Mixtures significantly increased forage dry matter yield by 61.39% and 1188.29% in the alfalfa–timothy mixture compared with alfalfa and timothy monocultures, respectively, and by 54.36% and 736.38% in the alfalfa–smooth bromegrass mixture compared with alfalfa and smooth bromegrass monocultures, respectively. Mixtures enhanced soil organic carbon, total nitrogen, nitrate nitrogen and ammonium nitrogen contents, and urease activity, but reduced microbial alpha diversity. Beneficial taxa, such as *Bacillus*, *Paenibacillus*, and *Mortierella*, were enriched. Soil nitrate nitrogen was identified as a key driver influencing bacterial functional composition, while soil organic carbon, ammonium nitrogen, water, alkaline phosphatase, and sucrase exhibited significant effects on fungal functional composition. This study demonstrated that alfalfa–grass mixtures enhance system productivity by improving soil physicochemical properties and reconstructing soil microbial community. It provides a theoretical basis from the viewpoint of soil health for establishing and managing sustainable cultivated grasslands.

## 1. Introduction

China possesses the world′s second-largest grassland area [1], which has long played a vital role in sustaining local livestock husbandry and ecological services. However, natural grassland productivity remains substantially low due to overgrazing [2] and climate change, constraining the development of the modern livestock industry. In this context, establishing cultivated grasslands has emerged as an efficient strategy to support sustainable livestock production [3,4]. Cultivated grassland can produce a great amount of high-quality forage, while this production is vulnerable and readily modulated by diverse factors such as soil health. Therefore, uncovering the mechanisms underlying the changing productivity will help a lot in optimizing the establishment and management of cultivated grassland.

Soil health serves as a critical determinant of forage production, directly influencing the productivity, stability, and sustainability of grassland ecosystems [5]. It is primarily characterized by two key indicators: soil microbial community and physicochemical properties [6,7]. Soil microorganisms drive the decomposition of soil organic matter and participate in the transformation of soil nitrogen (N) and phosphorus (P), regulating the availability of essential nutrients in the soil [8,9]. Furthermore, microbial metabolites enhance the stability of soil aggregates, subsequently optimizing soil physical structure [10]. Therefore, soil microorganisms are often described as the “engine” of terrestrial ecosystems, and maintaining the community structure is essential for sustaining soil fertility and plant productivity [11]. The diversity and stability of soil microbial communities are highly sensitive to environmental changes, manifesting as significant variations across vegetation systems [12]. Past studies have demonstrated that microbial diversity may be higher [13,14], lower [15], or barely change in diverse-species systems compared to in sole-species systems [16]. These underscore the potential to shape the soil microbiome through vegetation management such as changing grassland structure in cultivated grasslands. An appropriate species combination enhances surface coverage, root biomass, and exudate secretion [17,18]. These root-derived inputs actively reshape soil microbial community structure and function [19], which in turn ameliorates soil structure, strengthens nutrient retention, and boosts system productivity and stability [20]. As an important cultivation system, mixed intercropping grasslands (mixtures) leverage spatial complementarity to optimize resource use efficiency [21] with more species and more complex structures than monocultures. Mixture structure (i.e., species combination and mixing ratio) significantly affects productivity [22] and resource use [23,24]. However, the role of microorganisms in regulating resource use and production still remains uncovered in cultivated mixtures. It is therefore of great importance to investigate the community structure and function of microorganisms in diverse cultivated mixtures.

The legume–grass combination represents the most classical and widely adopted configuration of cultivated grasslands in practical applications [25]. Legumes may directly alter soil N cycling through biological N_2_ fixation (BNF) and influence related microbial functional groups [26], while grasses impact soil carbon (C) pool and physical structure with extensive and dense fibrous root systems [27]. The legume–grass combination thereby influences the microbial network structure of the rhizosphere microenvironment and even the entire soil matrix [28]. Yan et al. [28] demonstrated that an alfalfa (*Medicago sativa* L.)–*Elymus nutans* Griseb. mixture creates favorable conditions for microbial interactions, consequently promoting forage growth in the Tibetan Plateau. Luo et al. [29] found that, compared with oat monoculture soils, the soils of mixed cropping systems combining annual forage pea (*Pisum sativum* L.), common cornflower (*Vicia sativa* L.), or fava bean (*V. faba* L.) with oat (*Avena sativa* L.) have shown a higher relative abundance of dominant bacterial and fungal genera. The difference in growth and production of all species, especially between legume and grass, affects the return of organic matter and thus may regulate soil microorganism community, leading to various transformations of soil organic matter [30]. Clarifying how the community appears and its roles in diverse legume–grass mixtures has important theoretical significance for the cultivation and management of legume-containing grasslands through designing and optimizing species combinations. Additionally, the stand age of grasslands proves efficient in affecting the structure and function of the soil microbial community. For instance, Ma et al. [31] observed that the continuous cropping of alfalfa over six years led to detrimental succession in the rhizosphere microbial community. Therefore, it is critical to further understand the performance and mechanisms of the microbial community in perennial legume–grass mixtures during mid- to late-growth stages.

In this study, we hypothesized that different legume–grass combinations would lead to various soil physicochemical properties and microbial community characteristics, both of which should be closely coupled in the perennial legume–grass mixtures. Two mixing combinations were established using alfalfa and two grasses, timothy (*Phleum pratense* L.), and smooth bromegrass (*Bromus inermis* Leyss.), to systematically investigate the effects of species combination in aged mixture stands on soil physicochemical property and microbial community. Ultimately, this study aimed to elucidate (1) changes in soil microbiota and physicochemical properties with species combinations and (2) the coupling relationships between two soil health indicators in alfalfa–grass mixtures.

## 2. Materials and Methods

### 2.1. Experimental Site

**Figure 1:**
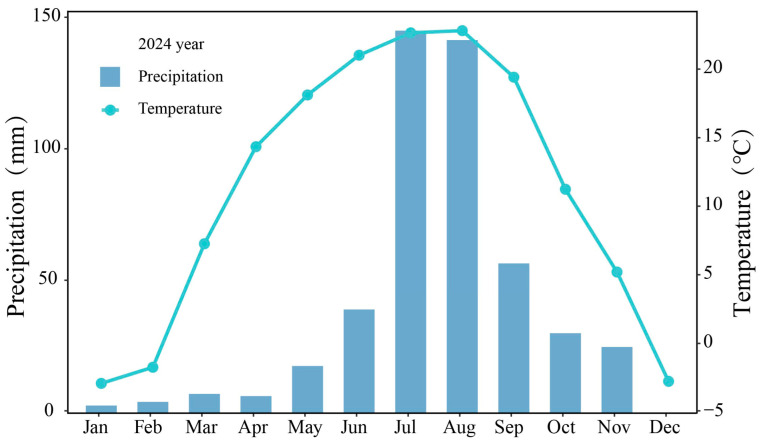
Monthly precipitation and monthly mean temperature at the experimental site during this study.

The field plots of this study were established at the Qingyang National Field Scientific Observation and Research Station of Grassland Agro-ecosystems (107°51′ E, 35°40′ N, altitude 1297 m) of Lanzhou University in September 2017. This study was conducted on the eight-year-old stands to analyze the characteristics of the soil and microbiome after seven years of experimentation. The climate is a temperate continental semi-arid climate. Long-term (1970–2024) mean annual precipitation is 543 mm, which falls mostly between July and September. The annual evaporation ranges from 1100 to 1500 mm. The mean annual temperature is 9.8 °C, and the frost-free period is 165 d. The monthly precipitation and monthly mean temperature during the experimental period are presented in Figure 1. The soil type is a Heilu soil (a calcic kastanozem), with a pH value ranging from 8.0 to 8.5. For 0–30 cm soil layer, organic C (SOC) content ranges between 6.36 and 7.15 g kg^−1^, total N (STN) content varies between 0.7 and 1.0 mg kg^−1^, nitrate N (NO_3_^−^, SNN) content varies between 17.3 and 23.3 mg kg^−1^, and ammonium N (NH_4_^+^, SAN) content changes between 2.6 and 3.0 mg kg^−1^. The preceding crop in the experimental fields was maize (*Zea mays* L.).

### 2.2. Experimental Design and Treatments

In this study, three forage crops including alfalfa, smooth bromegrass, and timothy, were used as experimental materials. Alfalfa is cultivated widely in arid and semi-arid areas in China, including the Loess Plateau. A local variety of alfalfa, *M. sativa* cv. Longdong, which has long been used in eastern Gansu Province, was used in this study. Smooth bromegrass, a perennial graminaceous species, performs well for its high nutritional value, palatability, stress tolerance, and utility in pasture establishment and sand fixation. Timothy, a perennial graminaceous species noted for its soft texture and favorable nutritional quality, is sown specifically in the eastern area of Gansu Province and sold as an excellent feed for racing horses.

The experiment employed a single-factor randomized complete block design. Two mixtures with different species combinations, alfalfa–smooth bromegrass (MB) and alfalfa–timothy (MP), were compared with three monocultures, alfalfa (M), smooth bromegrass (B), and timothy (P). There were three replicates for all five sowing patterns (treatments). There was a total of 15 plots and the size of each plot was 3 m × 3 m with a 0.5 m alley between plots. In each plot, ten rows of crops were planted with row space of 30 cm. The seeding rate for timothy and alfalfa was 15 kg ha^−1^ in monocultures, and for smooth bromegrass, 30 kg ha^−1^. For the mixtures, the two seeds were evenly sown in the same row with sowing ratios of 3:7 (alfalfa vs. grass), 5:5, and 7:3 [22]. That is, for example, when the sowing ratio was 3:7, the seeding rate of alfalfa would be 30% of the rate in monoculture and seeding rate of smooth brome or timothy would be 70% of the rates in monocultures. Note that in the subsequent calculation and analyses, the mean values of parameters for the mixtures were obtained from 3 sowing ratios to represent the value for MB or MP.

Referring to local practices, base fertilizer applications of 50 kg N ha^−1^ (urea, N ≥ 46%) and 60 kg P ha^−1^ (single superphosphate, P_2_O_5_ ≥ 16%) were applied prior to grassland establishment. No fertilizer was applied to the grasslands from 2017 to 2021, and from 2022, fertilizers of the same rates were applied annually at the green-returning stage. Conventional field management practices, such as weed and pest control, were carried out throughout the experimental period. No irrigation was applied during all seasons.

### 2.3. Plant and Soil Sampling

All the tests were conducted at the second cut of the eight year-old stands (2024). At the early flowering stage of alfalfa, two 50 cm crop fragments were sampled from each plot of all treatments. The samples were oven-dried at 105 °C for 30 min and then at 65 °C for 48 h. Dry matter weight was measured to determine forage dry matter yield (Y_DM_).

Soil sampling was conducted concurrently when crop samples were taken (the early flowering stage of alfalfa). Three soil cores of 0–20 cm were collected using a soil auger and combined into one composite sample for each plot. Immediately, the fresh soil samples were divided into two subsamples. One subsample was promptly sent out for high-throughput sequencing analysis. This analysis would help determine the structure and diversity of microorganisms. Another subsample was air-dried and then passed through a 2 mm sieve. This subsample would be used for determining soil physicochemical properties and enzyme activities.

### 2.4. Determination of Soil Physicochemical Properties

Soil pH was measured using a pH meter (Leici PHSJ-6L, Mettler Toledo, Greifensee, Switzerland) with a water-to-soil ratio of 1:5 [32]. Soil water content (SWC) was determined using the weighing method. SOC was determined using the potassium dichromate heating oxidation method with a total organic C analyzer (TOC, Elementar, Frankfurt, Germany). STN was measured using the Kjeldahl method [33] with a Kjeldahl auto-analyzer (Kjeltech 8400, Foss, Hilleroed, Denmark). STP and soil available P (SAP) were analyzed using a molybdenum-antimony colorimetric method [34] with a spectrophotometer (UV-2102 PCS, Shanghai Spectrum Instruments Co., Ltd., Shanghai, China). SNN and SAN were quantified using a fully automated discrete chemical analyzer (Smart Chem 450, AMS Alliance, Rome, Italy) [35]. The urease activity in the soil was measured using the indophenol blue colorimetric method. Specifically, the activities of alkaline phosphatase (APase) and sucrase were determined using commercial assay kits (Beijing Solarbio Science & Technology Co., Ltd., Beijing, China) [36].

### 2.5. High-Throughput Sequencing of Soil Microorganisms

Total genomic DNA was extracted from soil samples using the TGuide S96 Magnetic Bead Soil Genomic DNA Extraction Kit (Tiangen Biotech Co., Ltd., Beijing, China). The concentration of the extracted DNA was quantified with a microplate reader (Synergy HTX, Gene Company Limited, Shanghai, China) to standardize the template amount for subsequent amplification. Qualified genomic DNA was used as the template for target region amplification with specific primers. The near-full-length bacterial 16S rRNA gene was amplified using primers 27F (5′-AGRGTTTGATYNTGGCTCAG-3′) and 1492R (5′-TASGGHTACCTTGTTASGACTT-3′). The full-length fungal ITS region was amplified using primers ITS1F (5′-CTTGGTCATTTAGAGGAAGTAA-3′) and ITS4 (5′-TCCTCCGCTTATTGATATGC-3′). The amplified products were subjected to fragment analysis and library quantification for the target regions using the LabChip GX Touch system (Model: cls137031/E, PerkinElmer, Inc., Waltham, MA, USA). Libraries meeting the concentration criteria, as verified by the Qubit fluorescence quantification system, were enzymatically pre-complexed using the Revio Polymerase Kit, purified with Cleanup Beads, and sequenced on the Revio sequencer (Pacific Biosciences of California, Inc., Menlo Park, CA, USA) employing single-molecule real-time (SMRT) long-read sequencing technology (www.biocloud.net/).

### 2.6. Data Analysis

All statistical analyses and visualizations were performed in R version 4.3.3, primarily utilizing packages including ggplot2 [37], vegan [38], and agricolae [39]. Indicators such as forage yield, microbial abundance, and alpha diversity were first assessed for normality and homogeneity of variance. If the assumptions were met, one-way analysis of variance (ANOVA) followed by Duncan′s test was applied; otherwise, the Kruskal–Wallis test was used. Community structure was analyzed based on Bray–Curtis distances using principal coordinate analysis (PCoA) and non-metric multidimensional scaling (NMDS), with differences between groups evaluated by permutational multivariate analysis of variance (PERMANOVA) using 999 permutations. Microbial functions were predicted using PICRUSt2 (for bacteria) and FUNGuild (for fungi). The overall relationships between microbial functions and soil physicochemical properties were examined by the Mantel test, and Spearman correlation analysis was employed to investigate associations between soil physicochemical properties and microbial functions.

## 3. Results

### 3.1. Forage Dry Matter Yield

**Figure 2:**
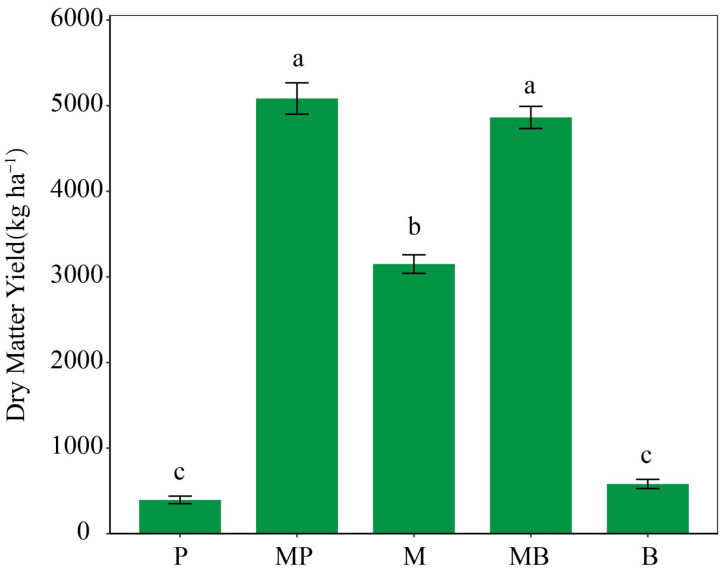
Forage biomass yield in monocultures and mixtures of various species combinations. P, timothy monoculture; MP, alfalfa–timothy mixture; M, alfalfa monoculture; MB, alfalfa–smooth bromegrass mixture; B, smooth bromegrass monoculture. Different lowercase letters indicate significant differences (*p* < 0.05) among sowing patterns.

Analysis of variance indicated that sowing pattern significantly (*p* < 0.05) affected forage Y_DM_ (Figure 2). MP and MB mixtures produced significantly higher Y_DM_ (*p* < 0.05) than monocultures, and M monoculture produced significantly higher Y_DM_ (*p* < 0.05) than grass monocultures. MP mixture increased Y_DM_ by 61.39% and 1188.29% compared with M and P monocultures, respectively, while MB mixture increased Y_DM_ by 54.36% and 736.38% compared with M and B monocultures, respectively. No significant difference was observed between MP and MB mixtures. In MP and MB mixtures, Y_DM_ were 5084 and 4863 kg ha^−1^, respectively.

### 3.2. Soil Physicochemical Properties

Compared with corresponding grass monocultures, legume–grass mixing significantly (*p* < 0.05) improved soil physicochemical properties (Table 1). Compared with B monoculture, MB mixture significantly (*p* < 0.05) increased SOC, STN, SNN, SAN, SWC, and urease activity by 22.05%, 12.00%, 86.18%, 19.72%, 13.77%, and 200.84%, respectively, but significantly (*p* < 0.05) decreased soil pH by 1.77%. There were few differences between MB mixture and M monoculture, except that soil pH and urease activity were significantly higher (*p* < 0.05) in MB mixture. Compared with P monoculture, MP mixture enhanced SOC, STN, SNN, SAN, and SWC by 33.13%, 4.17%, 47.06%, 13.07%, and 4.64%, respectively. No significant differences were observed between MP mixture and M monoculture for most of the measured soil parameters. Most of the soil physicochemical properties tended to be higher in MB mixture than MP mixture, while there were few differences between the two grass monocultures. Compared with grass monocultures, M monoculture had higher SOC, SNN, SAN, and SWC, and lower soil pH. Overall, alfalfa mixtures led to significant changes in SOC, soil N nutrient contents and urease activity, while soil P nutrient contents and APase activity were affected less.

Most soil physicochemical properties reached their highest levels in mixtures. In MB mixture, SOC, STN, SNN, SAN, SWC, and urease activity were 2.38 kg m^−2^, 0.28 kg m^−2^, 6.33 g m^−2^, 1.70 g m^−2^, 12.97%, and 553.37 mg m^−2^ d^−1^, respectively. In MP mixture, SOC, STN, SNN, SAN, and SWC were 2.21 kg m^−2^, 0.25 kg m^−2^, 4.75 g m^−2^, 1.73 g m^−2^, and 12.40%, respectively.

**Table 1:** Soil physicochemical properties in monocultures and mixtures of various species combinations.

Soil Physicochemical Property	B	MB	M	MP	P
SOC (kg m^−2^)	1.95 ± 0.08 ^b^	2.38 ± 0.08 ^a^	2.35 ± 0.11 ^a^	2.21 ± 0.04 ^ab^	1.66 ± 0.06 c
STN (kg m^−2^)	0.25 ± 0.01 ^b^	0.28 ± 0.01 ^a^	0.25 ± 0.01 ^ab^	0.25 ± 0.00 ^ab^	0.24 ± 0.00 ^b^
SNN (g m^−2^)	3.40 ± 0.04 ^b^	6.33 ± 0.89 ^a^	5.63 ± 0.30 ^ab^	4.75 ± 0.28 ^ab^	3.23 ± 0.57 ^b^
SAN (g m^−2^)	1.42 ± 0.06 ^b^	1.70 ± 0.06 ^a^	1.63 ± 0.01 ^a^	1.73 ± 0.04 ^a^	1.53 ± 0.09 ^ab^
STP (kg m^−2^)	0.23 ± 0.00 ^a^	0.23 ± 0.00 ^a^	0.23 ± 0.01 ^a^	0.22 ± 0.01 ^a^	0.24 ± 0.01 ^a^
SAP (g m^−2^)	1.36 ± 0.02 ^a^	1.53 ± 0.06 ^a^	1.50 ± 0.15 ^a^	1.44 ± 0.14 ^a^	1.45 ± 0.07 ^a^
Soil pH	8.04 ± 0.03 ^a^	7.90 ± 0.01 ^b^	7.74 ± 0.05 c	7.96 ± 0.03 ^ab^	7.92 ± 0.07 ^ab^
SWC (%)	11.40 ± 0.19 ^b^	12.97 ± 0.28 ^a^	12.70 ± 0.57 ^a^	12.40 ± 0.22 ^ab^	11.85 ± 0.64 ^ab^
Urease (mg m^−2^ d^−1^)	183.94 ± 1.48 ^b^	553.37 ± 11.77 ^a^	193.18 ± 13.95 ^b^	165.30 ± 9.27 ^b^	190.09 ± 7.57 ^b^
APase (mmol m^−2^ d^−1^)	2.50 ± 0.15 ^a^	3.20 ± 0.21 ^a^	2.59 ± 0.19 ^a^	2.82 ± 0.18 ^a^	2.97 ± 0.10 ^a^
Sucrase (g m^−2^ d^−1^)	22.52 ± 3.25 ^a^	20.70 ± 1.29 ^a^	21.14 ± 0.37 ^a^	21.91 ± 0.99 ^a^	22.80 ± 3.53 ^a^

Values are means ± s.e. P, timothy monoculture; MP, alfalfa–timothy mixture; M, alfalfa monoculture; MB, alfalfa–smooth bromegrass mixture; B, smooth bromegrass monoculture. SOC, soil organic C; STN, soil total N; SNN, soil nitrate N; SAN, soil ammonium N; STP, soil total P; SAP, soil available P; SWC, soil water content; APase, alkaline phosphatase. Different lowercase letters represent significant differences (*p* < 0.05) among sowing patterns.

**Figure 3:**
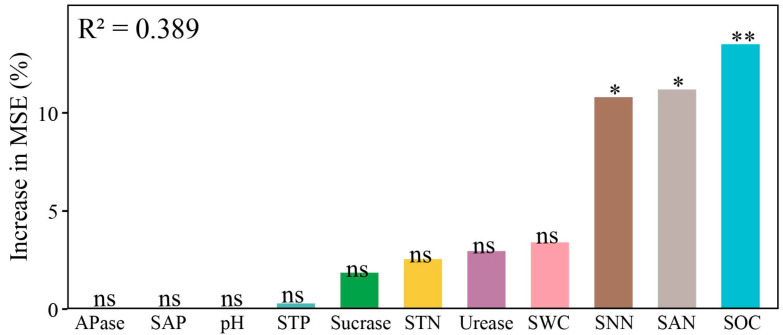
Mean predictive importance of each indicator for dry matter yield presented by MSE increase. SOC, soil organic C; STN, soil total N; SNN, soil nitrate N; SAN, soil ammonium N; STP, soil total P; SAP, soil available P; SWC, soil water content; APase, alkaline phosphatase. *, *p* < 0.05; **, *p* < 0.01; ns, not significant.

The random forest analysis of mean predictive importance for Y_DM_ identified SOC, SNN, and SAN as significant predictors (*p* < 0.05; Figure 3). Notably, SOC demonstrated a highly significant influence (*p* < 0.01) on Y_DM_. In contrast, the remaining indicators did not exhibit significant effects on Y_DM_. These suggested that SOC and N nutrients played a more active role in supporting mixing effects.

### 3.3. Diversity of Bacterial and Fungal Communities

#### 3.3.1. Alpha Diversity

**Figure 4:**
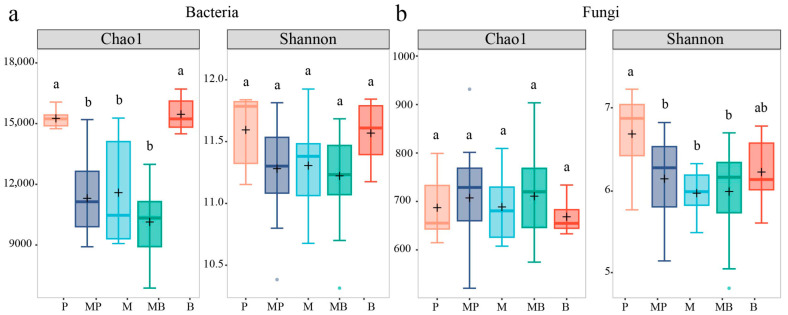
Alpha diversity of (**a**) soil bacterial and (**b**) fungal communities. P, timothy monoculture; MP, alfalfa–timothy mixture; M, alfalfa monoculture; MB, alfalfa–smooth bromegrass mixture; B, smooth bromegrass monoculture. Different lowercase letters represent significant differences (*p* < 0.05) among sowing patterns.

Analyses of Chao1 and Shannon indices indicated significant differences in the alpha diversity of soil bacterial and fungal communities (*p* < 0.05) among sowing patterns (Figure 4). For bacterial community, the Chao1 index was significantly higher in B and P monocultures than other patterns, while the Shannon index tended to be higher in B and P monocultures, even if no significant differences were observed across patterns (Figure 4a). For fungal community, the Chao1 index tended to be less in B and P monocultures even if no significant differences were detected among sowing patterns, whereas the Shannon index was significantly higher (*p* < 0.05) in B and P monocultures compared with other patterns (Figure 4b). Overall, these suggested that sole grass cropping significantly increased the richness of rare bacteria while also enhancing the evenness of the fungal community compared with mixed cropping.

#### 3.3.2. Beta Diversity

**Figure 5:**
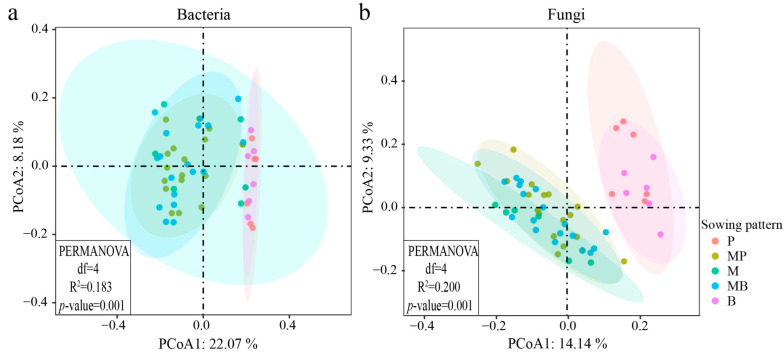
Beta diversity of (**a**) bacterial and (**b**) fungal communities. P, timothy monoculture; MP, alfalfa–timothy mixture; M, alfalfa monoculture; MB, alfalfa–smooth bromegrass mixture; B, smooth bromegrass monoculture.

Bray–Curtis distance matrices were used for principal coordinate analysis (PCoA) and PERMANOVA tests demonstrating the beta diversity of soil organisms (Figure 5). For soil bacterial community (Figure 5a), the cumulative contribution rate of PCoA1 and PCoA2 was 30.25%, with PCoA1 accounting for 22.07% of the variation. MP and MB mixtures were clearly separated from P and B monocultures, and were relatively distant along the PCoA1 axis. PERMANOVA tests further confirmed significant differences in microbial community structure (R^2^ = 0.183, *p* = 0.001) among sowing patterns, although no significant difference was observed between M monoculture and the other four patterns. For soil fungal community (Figure 5b), the cumulative contribution rate of PCoA1 and PCoA2 was 23.47%, with PCoA1 explaining 14.14% of the variation. The two mixtures and M monoculture were distinctly separated from P and B monocultures, exhibiting considerable separation along the PCoA1 axis. PERMANOVA tests also indicated significant differences in microbial community structure (R^2^ = 0.200, *p* = 0.001) among sowing patterns. Overall, the community compositions (beta diversity) of both bacteria and fungi were significantly influenced by sowing patterns. Notably, P and B monocultures formed distinct clusters that were clearly separated from the mixtures along the primary ordination axis, indicating that mixed cropping has led to significant reconstruction of soil microbial assemblages.

### 3.4. Composition of Bacterial and Fungal Communities

#### 3.4.1. Composition at the Phylum Level

To analyze the taxonomic composition of bacterial and fungal communities across sowing patterns, full-length sequencing of the bacterial 16S rRNA gene and the fungal internal transcribed spacer (ITS) region was performed using the PacBio sequencing platform. Analysis of the 16S data yielded 3,320,968 circular consensus sequencing reads, while the ITS data produced 3,226,912 high-quality sequences. Consequently, 34,207 bacterial operational taxonomic units (OTUs) were identified, and 2843 fungal OTUs were obtained.

**Figure 6:**
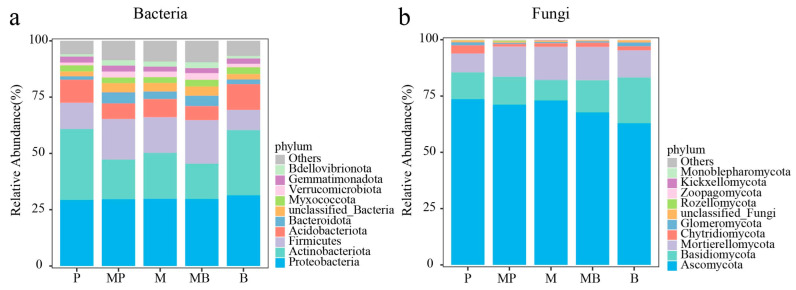
Relative abundance of microbial taxa at the phylum level. (**a**) Bacteria; (**b**) fungi. P, timothy monoculture; MP, alfalfa–timothy mixture; M, alfalfa monoculture; MB, alfalfa–smooth bromegrass mixture; B, smooth bromegrass monoculture.

The dominant bacterial phyla across all patterns were *Proteobacteria* (26.80–30.73%), *Actinobacteriota* (8.60–18.22%), *Acidobacteriota* (7.15–11.68%), *Firmicutes*, *Bacteroidota*, *unclassified_Bacteria*, *Myxococcota*, *Verrucomicrobiota*, *Gemmatimonadota*, and *Bdellovibrionota*, with *Proteobacteria* being the most abundant (Figure 6a). Compared with grass monocultures, the two mixtures and M monoculture increased the relative abundance of *Firmicutes*, but reduced *Actinobacteriota*. Compared with M monoculture, the two mixtures increased *Firmicutes* more and reduced *Actinobacteriota* more. It also suggested that alfalfa would have played a vital role in reconstructing the composition of bacteria at the phylum level after intercropping with grasses.

For fungal community, *Ascomycota* (62.90–73.56%), *Basidiomycota* (9.11–20.37%), and *Mortierellomycota* (8.38–14.85%) represented the majority of the community (Figure 6b). Compared with P monoculture, MP mixture increased the relative abundance of *Mortierellomycota*, but reduced *Ascomycota*. Compared with M monoculture, MP mixture increased the relative abundance of *Basidiomycota*, but reduced *Ascomycota*. In contrast, MB mixture and M monoculture increased the relative abundances of *Ascomycota* and *Mortierellomycota* compared with B monoculture, but reduced *Basidiomycota*. Compared with M monoculture, MB mixture increased *Ascomycota* less and reduced *Basidiomycota* less. It also suggested that alfalfa would have played different roles in reconstructing the composition of fungi at the phylum level after intercropping with grasses.

#### 3.4.2. Composition at the Genus Level

**Figure 7:**
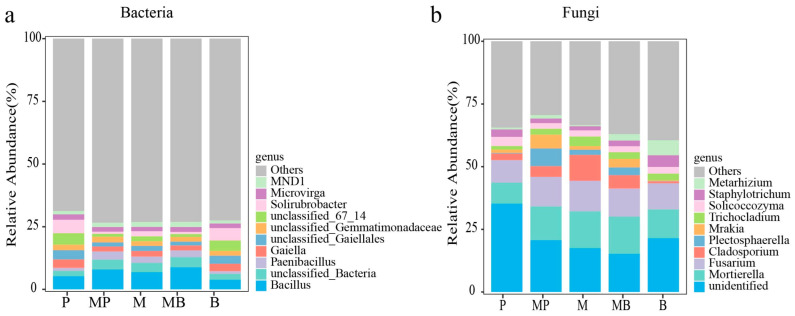
Relative abundance of microbial taxa at the genus level. (**a**) Bacteria; (**b**) fungi. P, timothy monoculture; MP, alfalfa–timothy mixture; M, alfalfa monoculture; MB, alfalfa–smooth bromegrass mixture; B, smooth bromegrass monoculture.

At the bacterial genus level, *Bacillus* (3.87–8.85%), *unclassified_Bacteria* (2.10–4.06%), *Paenibacillus* (1.08–3.26%), *Gaiella*, and *unclassified_Gaiellales* exhibited the highest relative abundances (Figure 7a). Compared to grass monocultures, MP and MB mixtures and M monoculture increased the relative abundances of *Bacillus*, *unclassified_Bacteria*, and *Paenibacillus*, but reduced *Gaiella* and *unclassified_Gaiellales*. Compared with M monoculture, two mixtures increased *Bacillus*, *unclassified_Bacteria*, and *Paenibacillus* more, but reduced *Gaiella* and *unclassified_Gaiellales* more. These also suggested that alfalfa would have played a vital role in reconstructing the composition of bacteria at the genus level after intercropping with grasses.

For fungal community, *unidentified* (15.28–35.33%), *Mortierella* (8.30–14.83%), *Fusarium* (9.01–12.18%), and *Cladosporium* were the dominant genera across all sowing patterns (Figure 7b). Compared with grass monocultures, MP and MB mixtures and M monoculture increased the relative abundances of *Mortierella*, *Fusarium*, *Cladosporium*, and *Plectosphaerella*, but decreased *unidentified*. Compared with M monoculture, two mixtures increased *Plectosphaerella* more and increased *Cladosporium* less, while MP mixture decreased *unidentified* less and MB mixture reduced *unidentified* more. These also suggested that alfalfa would have played contrasting roles in reconstructing the composition of fungi at the genus level after intercropping with grasses.

### 3.5. Functional Profile Prediction and Its Link with Soil Physicochemical Property

**Figure 8:**
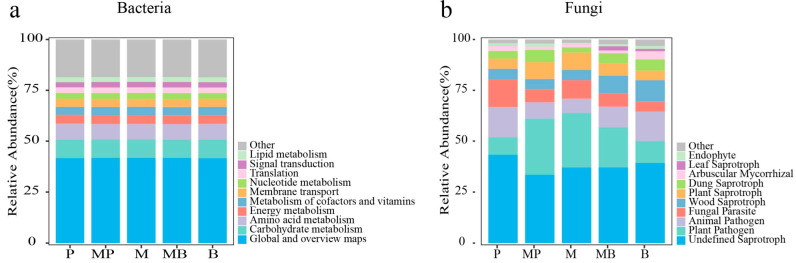
Functional profile prediction based on the relative abundance of microbial taxa at the genus level. (**a**) Bacteria; (**b**) fungi. P, timothy monoculture; MP, alfalfa–timothy mixture; M, alfalfa monoculture; MB, alfalfa–smooth bromegrass mixture; B, smooth bromegrass monoculture.

Functional prediction of bacterial and fungal communities using PICRUSt2 and FUNGuild indicated that microbial functional composition was closely associated with nutrient cycling and energy flow (Figure 8). Bacterial functional profile was dominated by core metabolic processes, with Global and Overview Maps (41.59–41.86%) being the most abundant category, followed by carbohydrate metabolism (9.05–9.15%) and amino acid metabolism (7.55–7.73%), collectively accounting for over 65% of the predicted functions (Figure 8a). However, no significant differences were observed in the relative abundance of functional bacteria among sowing patterns. Fungal community was primarily composed of saprotrophic and pathogenic guilds, with undefined saprotrophs (33.45–43.26%) being the most abundant, followed by plant pathogen (8.73–27.64%) and animal pathogen (7.08–14.79%) (Figure 8b). Additionally, the relative abundance of plant pathogen and animal pathogen exceeded 20%. The relatively high abundances of wood saprotroph and plant saprotroph further suggested that fungal community would likely play a critical role in plant residue decomposition. Compared with P monoculture, MP mixture and M monoculture increased the relative abundances of plant pathogen and plant saprotroph, but decreased undefined saprotroph, animal pathogen, and fungal parasite. Compared with M monoculture, MP mixture decreased undefined saprotroph and fungal parasite more, and decreased animal pathogen less. Compared with B monoculture, MB mixture and M monoculture increased the relative abundances of plant pathogen, fungal parasite, and plant saprotroph, but decreased undefined saprotroph, animal pathogen, wood saprotroph, and dung saprotroph. Compared with M monoculture, MB mixture increased plant pathogen, fungal parasite, and plant saprotroph less, but decreased animal pathogen, wood saprotroph, and dung saprotroph more. These also suggested that alfalfa would have played no role in modulating the functional profile of bacteria at the genus level after intercropping with grasses, while there would be contrasting effects in modulating the functional profile of fungi.

**Figure 9:**
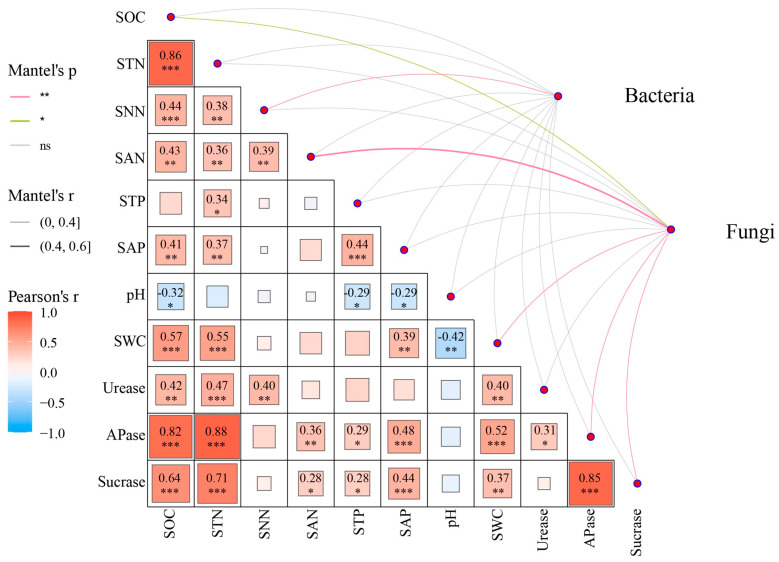
Correlations among soil physicochemical properties and their links with predicted microbial functional profiles. SOC, soil organic C; STN, soil total N; SNN, soil nitrate N; SAN, soil ammonium N; STP, soil total P; SAP, soil available P; SWC, soil water content; APase, alkaline phosphatase. *, ** and *** show significant correlations at the levels of 0.05, 0.01 and 0.001, respectively. ns means no significance.

Mantel test results indicated significant correlations between soil physicochemical properties and microbial functional composition (Figure 9). SNN was identified as a key factor significantly influencing bacterial functional composition, while SOC, SAN, SWC, soil APase, and sucrase exhibited significant effects on fungal functional composition. In addition, correlation analysis revealed significantly positive correlations among SOC, STN, SNN, SAN, STP, SAP, urease, APase, and sucrase, if any (Figure 9). In contrast, soil pH exhibited significantly negative correlations with SOC, STP, SAP, and SWC.

## 4. Discussion

### 4.1. Effect of Sowing Pattern on Forage Yield

The adoption of mixed intercropping systems is recognized for its ability to exploit spatial complementarity and optimize resource use efficiency, thereby enhancing forage biomass production [21]. Among the monocultures in this study, alfalfa (M) yielded the highest Y_DM_, which is consistent with its adaptability and vigorous growth [40]. This made it readily and advantageous when intercropped with other crops. In this study, both legume–grass mixtures (MP and MB) significantly surpassed all monocultures, increasing Y_DM_ by 61.39% and 1188.29% (for MP), 54.36% and 736.38% (fog MB) compared with M monoculture or corresponding grass monoculture. However, the two combinations should share contrasting mechanisms contributing to these advantages. MP mixture likely enhances nutrient use efficiency, mainly through the complementary resource acquisition of efficient P uptake by timothy coupled with strong BNF by alfalfa [41]. In contrast, MB mixture′s advantage may result from the extensive root system of smooth bromegrass improving soil structure and water retention, thereby facilitating alfalfa growth [27]. The complementary mechanism exists in broader research. For instance, Bi et al. [42] found that alfalfa–cocksfoot (*Dactylis glomerata* L.) intercropping outperformed sainfoin (*Onobrychis viciifolia* L.)–tall fescue (*Festuca arundinacea* Schreb.) intercropping in terms of resource acquisition. This superiority is primarily attributed to the stronger competitive ability and higher ecological niche of the former combination, whereas the latter is constrained by factors such as the poor tolerance of sainfoin to frequent cutting and the relatively low plant height of tall fescue, resulting in lower overall resource use efficiency.

### 4.2. Effects of Sowing Pattern on Soil Physicochemical Properties

A consensus has emerged from numerous studies that mixed intercropping can markedly improve soil physicochemical conditions, which leads to an increase in the pool of plant available nutrients [43,44]. In this study, mixtures significantly increased the contents of SOC, STN, SNN, and SAN, which is consistent with previous findings [25,45]. This improvement can be attributed to several reasons. Firstly, the strong BNF of alfalfa should have helped enhance the N sources for alfalfa–grass mixtures [26,46]. In these mixtures, grass exhaustion of soil mineral N also helps promote BNF of alfalfa. Secondly, alfalfa–grass mixture significantly enriched bacterial taxa with urease-hydrolyzing capabilities, primarily from the genera *Bacillus* and *Paenibacillus* [47,48]. This was evidenced by the increased relative abundances of these bacteria in two mixtures. Both bacteria possess strong urease-producing capabilities [49,50,51], thus playing a key role in N cycling for the mixtures. Thirdly, the improved soil N nutrients consequently led to promoted forage biomass production. The higher biomass production in mixtures also implies greater return of plant organic matter into the soil, increasing SOC and “raw material” for decomposition by microorganisms. Finally, the enhanced root exudate secretion due to the multiple species in the systems, together with increased organic matter return, would continuously stimulate microbial activity, accelerate organic matter mineralization, and release more available nutrients [52,53]. Intriguingly, no significant differences were observed in soil P nutrient contents between mixtures and monocultures. This was in accordance with similar soil APase activity among sowing patterns, suggesting mixed intercropping failed to change the mechanisms involved in P release from plant organic matter. These details still need extensive investigation.

Soil N nutrient-related processes may be primarily attributed to the enrichment of specific functional microbial communities, i.e., *Bacillus* and *Paenibacillus*. These microorganisms showed a positive correlation with the increase in urease activity [47,48]. However, MB mixture in this study significantly enhanced soil urease activity, while MP mixture tended to decrease soil urease activity. The contrasting results may be partly explained by the difference in fungal composition between two mixtures. There were significantly higher abundances of *Plectosphaerella* and *Mrakia* in MP mixture than MB mixture. *Plectosphaerella* contains known plant-pathogenic species, whose activity can impair root health and alter rhizosphere microenvironments [54,55], potentially creating unfavorable conditions for urease-producing bacteria (e.g., *Bacillus* and *Paenibacillus*). Similarly, *Mrakia*, a psychrotolerant yeast genus, may indicate niches with lower metabolic activity that are suboptimal for urease function [56]. Therefore, the enrichment of these fungal taxa in the MP mixture could potentially suppress the abundance or activity of key urease-producing bacterial communities, leading to the observed decrease in urease activity. However, the precise mechanistic interactions warrant further investigation. Additionally, SWC was significantly positively correlated with enzyme activity, i.e., urease, APase and sucrase activities. This finding aligned with established knowledge that soil moisture is a critical factor regulating enzyme activities [57]. The improved water regime likely creates a more favorable microenvironment for both microbial proliferation and enzymatic reactions. However, SWC varied among sowing patterns, but failed to change consistently with enzyme activities. Therefore, the changing profiles of enzyme activities, especially contrasting urease activity in MP and MB mixtures, likely result from the combined effects of multiple factors including SWC, soil pH, soil microorganism, etc.

### 4.3. Effects of Sowing Pattern on Soil Microbial Community Structure and Function

Numerous studies have demonstrated that plant species and cropping system significantly influence soil microbial community structure and function [28,58,59]. In this study, alfalfa–grass mixtures significantly reduced the bacterial Chao1 index and fungal Shannon index, and differed significantly from grass monocultures with contrasting microbial community compositions. These results were partly consistent with Zhao et al. [15], who reported that intercropping can reduce bacterial richness. The reduction in alpha diversity indicated that the microbiome in the mixtures was shifting towards a more specialized and functionally optimized state. In the mixtures, plant community appears to selectively recruit a less diverse but more cooperative microbial consortium that is highly efficient in processing the specific substrates available in soils [60]. This functional specialization was also corroborated by composition changes in bacterial and fungal communities. At the bacterial phylum level, mixed intercropping significantly increased the relative abundance of *Firmicutes* but decreased *Actinobacteriota*. This shift may be associated with divergent C sources derived from root exudates of different plant species in the mixture [61]. For fungal community, the phylum *Mortierellomycota* was significantly enriched in mixtures. This type of fungi possesses the capability to decompose plant residues and provide raw materials for humus synthesis [62]. Their enrichment is likely closely linked to the elevated soil-available N and SOC contents in mixtures [53]. At the genus level, mixed intercropping significantly increased the relative abundances of bacteria including *Bacillus*, *unclassified_Bacteria*, *Paenibacillus*, *Gaiella*, etc., and fungi including *Mortierella*, *Fusarium*, *Cladosporium*, *Plectosphaerella*, etc. Some strains of *Bacillus* are known to secrete urease and promote N mineralization [49,50], aligning with the enhanced urease activity observed in this study. Some *Mortierella* strains have been significantly correlated with soil organic matter content [63].

However, we failed to observe significant differences in the relative abundance of specific functional bacteria which were highly involved in core metabolic processes essential for nutrient cycling and energy flow, such as carbohydrate and amino acid metabolism. In contrast, abundant wood, plant and dung saprotrophs were observed in mixtures, demonstrating a close link between fungal function shift and soil nutrient enhancement [64]. The high abundances of wood and plant saprotroph groups indicated that fungal community plays a key role in plant residue decomposition [65]. Furthermore, this study revealed that in aging alfalfa–grass mixtures, soil fungal community became increasingly dominated by saprotrophic and pathogenic guilds, with the relative abundance of plant and animal pathogens exceeding 20%. This shift may contribute to grassland degradation over time. The increased pathogen abundance was likely driven by concurrent shifts in soil properties (e.g., SOC, nitrogen) and plant composition under specific mixtures, potentially triggering negative feedback loops that accelerate degradation processes [66]. The optimized microbial community structure, characterized by reduced diversity but heightened core metabolic activity, is a key biological mechanism underpinning the enhanced ecosystem functioning observed in the mixtures.

## 5. Conclusions

Alfalfa–grass mixtures significantly improved soil physicochemical properties and optimized the structure of the soil microbial community. Specifically, the mixtures could recruit a large number of soil-beneficial bacteria and fungi, including *Bacillus*, *Paenibacillus*, and *Mortierella*, favoring increases in the contents of soil organic carbon, total nitrogen, nitrate nitrogen, and ammonium nitrogen, as well as soil urease activity. These improvements in soil health indicators, particularly soil organic carbon, nitrate nitrogen, and ammonium nitrogen, were closely linked to enhanced forage yield, suggesting these factors and associated microbial communities might be key predictors of system productivity. These changes collectively improved the growth environment and promoted forage production. No significant differences were observed between the two alfalfa–grass combinations in most cases, demonstrating that positive feedback occurred regardless of the specific grass species paired with alfalfa. This indicated that the legume–grass interaction per se, rather than a particular combination, is the primary driver of system productivity enhancement. The findings provide a scientific basis for dynamic plant–microbe–soil feedback for guiding sustainable pasture establishment and management.

## Data Availability

The original contributions presented in this study are included in the article. Further inquiries can be directed to the corresponding author.
